# *Portiera* Gets Wild: Genome Instability Provides Insights into the Evolution of Both Whiteflies and Their Endosymbionts

**DOI:** 10.1093/gbe/evaa216

**Published:** 2020-10-13

**Authors:** Diego Santos-Garcia, Natividad Mestre-Rincon, David Ouvrard, Einat Zchori-Fein, Shai Morin

**Affiliations:** Department of Entomology, The Robert H. Smith Faculty of Agriculture, Food and Environment, The Hebrew University of Jerusalem, Rehovot, Israel; Department of Entomology, The Robert H. Smith Faculty of Agriculture, Food and Environment, The Hebrew University of Jerusalem, Rehovot, Israel; Department of Life Sciences, Natural History Museum, London, United Kingdom; Entomology and Invasive Plants Unit, Plant Health Laboratory, ANSES, Montferrier-sur-Lez, France; Department of Entomology, Newe-Ya’ar Research Center, ARO, Ramat-Yishai, Israel; Department of Entomology, The Robert H. Smith Faculty of Agriculture, Food and Environment, The Hebrew University of Jerusalem, Rehovot, Israel

**Keywords:** divergence dating, genome stasis, long-enduring taxon, molecular evolution, symbiosis, whitefly development, whitefly systematics

## Abstract

Whiteflies (Hemiptera: Sternorrhyncha: Aleyrodidae) are a superfamily of small phloem-feeding insects. They rely on their primary endosymbionts "*Candidatus* Portiera aleyrodidarum" to produce essential amino acids not present in their diet. *Portiera* has been codiverging with whiteflies since their origin and therefore reflects its host’s evolutionary history. Like in most primary endosymbionts, the genome of *Portiera* stays stable across the Aleyrodidae superfamily after millions of years of codivergence. However, *Portiera* of the whitefly *Bemisia tabaci* has lost the ancestral genome order, reflecting a rare event in the endosymbiont evolution: the appearance of genome instability. To gain a better understanding of *Portiera* genome evolution, identify the time point in which genome instability appeared and contribute to the reconstruction of whitefly phylogeny, we developed a new phylogenetic framework. It targeted five *Portiera* genes and determined the presence of the DNA polymerase proofreading subunit (*dnaQ*) gene, previously associated with genome instability, and two alternative gene rearrangements. Our results indicated that *Portiera* gene sequences provide a robust tool for studying intergenera phylogenetic relationships in whiteflies. Using these new framework, we found that whitefly species from the *Singhiella*, *Aleurolobus*, and *Bemisia* genera form a monophyletic tribe, the Aleurolobini, and that their *Portiera* exhibit genome instability. This instability likely arose once in the common ancestor of the Aleurolobini tribe (at least 70 Ma), drawing a link between the appearance of genome instability in *Portiera* and the switch from multibacteriocyte to a single-bacteriocyte mode of inheritance in this tribe.

Significance

Whiteflies have established a mutualistic relationship with *Portiera aleyrodidarum*, a symbiotic bacterium. A long history of strict mother-to-offspring transmission of *Portiera* allows this symbiont to reflect well its host evolutionary history. Moreover, *Portiera* genomes usually show high synteny, but in rare cases, genomic instability is present. As the current molecular and morphological classification tools for whiteflies are limited and prone to significant errors, we used the unique characteristics of *Portiera* genomes to study both *Portiera* and whitefly evolution. This framework allowed us to propose a new working hypothesis for the evolution of the rare genomic instability in *Portiera*, involving a switch from multi- to a single-bacteriocyte mode of inheritance in whiteflies.

## Introduction

Whiteflies are small phloem-feeding insects, which, together with aphids, scale insects, and psyllids, form the Sternorrhyncha suborder (Grimaldi and Engel 2005). Whiteflies are classified into one superfamily the Aleyrodoidea that includes one family, the Aleyrodidae. The Aleyrodidae consist of three extant subfamilies, the Udamoselinae, the Aleurodicinae, and the Aleyrodinae, and an extinct one, the Bernaeinae. The Udamoselinae subfamily contains only one genus and two species. The Aleurodicinae subfamily contains 21 extant genera, mainly distributed in Neotropical/Australasian regions (Charles 2010; Ouvrard and Martin 2020). The Aleyrodinae, with at least 142 described genera, is the most diverse and globally distributed subfamily and includes the major pest species *Bemisia tabaci* and *Trialeurodes vaporariorum* (Manzari and Quicke 2006; Ouvrard and Martin 2020). Although the extant whitefly subfamilies were reported to originate in the Middle Cretaceous (Campbell et al. 1994), the first fossils of the Aleurodicinae and Aleyrodinae subfamilies were dated to the Lower Cretaceous (Drohojowska and Szwedo 2015). During that period, whiteflies were associated with gymnosperm forests and/or proangiosperms, in contrast to extant whitefly species, which feed mainly on angiosperms. It is assumed that the emergence of angiosperms in the Lower Cretaceous opened new environmental niches and has promoted diversification and speciation of whiteflies along with their angiosperm hosts (Middle–Upper Cretaceous), leading to the emergence of the modern whitefly species (Drohojowska and Szwedo 2015).

Whiteflies, as most sternorrhynchan insects, harbor obligatory intracellular bacterial symbionts (P-endosymbionts) within specialized cells, termed bacteriocytes. Generally, these P-endosymbionts complement the restricted diets of their hosts (plant sap) and possess genomes reduced to a basic set of genes devoted to maintaining the symbiotic relationship (e.g., essential amino acids biosynthesis) and minimal cell functions (Hansen and Moran 2014; Latorre and Manzano-Marín 2017). The P-endosymbiont of whiteflies is “*Candidatus* Portiera aleyrodidarum” (hereafter *Portiera*) (Thao and Baumann 2004), which forms a monophyletic clade with “*Ca.* Carsonella ruddii”, the P-endosymbiont of psyllids. Based on molecular data, it has been proposed that the ancestral symbiosis was established in the Psyllinea lineage (Shcherbakov 2000), before its divergence into the Aleyrodoidea and Psylloidea lineages (Santos-Garcia et al. 2014). Because *Portiera*, as other P-endosymbionts, exhibits strict mother-to-offspring transmission, it has been codiverging with whiteflies since their origin. Moreover, no host-switching of *Portiera* has been documented (Thao and Baumann 2004; Santos-Garcia et al. 2015), even among recently diverged species belonging to the same species complex (Hsieh et al. 2014; Wang et al. 2019). Therefore, *Portiera* lineages reflect well both their own and their hosts phylogenetic relationships (De Vienne et al. 2013) and divergence times (Santos-Garcia et al. 2015).

Until present, only three *Portiera* genomes from species others than *B. tabaci* have been sequenced: *Aleurodicus dispersus* and *Aleurodicus floccissimus* from the Aleurodicinae subfamily, and *T. vaporariorum* from the Aleyrodinae. Like other P-endosymbionts, these three *Portiera* have maintained a genome stasis since the emergence of both the Aleurodicinae and the Aleyrodinae whitefly subfamilies, more than 135 Ma (Sloan and Moran 2013; Santos-Garcia et al. 2015). In contrast, *Portiera* genomes from the *B. tabaci* species complex, although syntenic among themselves, are extensively rearranged when compared with the other three published *Portiera* genomes. The genome rearrangements of *Portiera* from *B. tabaci* seem to be correlated with a massive loss of genes required for correct DNA replication and the repair machinery. These losses include the DNA polymerase III subunit epsilon *dnaQ*, which is required for repairing spontaneous mutations (proofreading activity) (Sloan and Moran 2013; Santos-Garcia et al. 2015). Extensive rearrangements are very uncommon events in P-endosymbionts evolution (Moran and Bennett 2014), and therefore, it is not clear if the genome instability of *Portiera* from *B. tabaci* is a unique event or a more general phenomenon present in other related and unrelated *Portiera* lineages.

In this work, we aimed to deepen our understanding of *Portiera* genome evolution and the origin of genome instability. Because P-endosymbionts gene sequences have been recognized as a valuable resource for reconstructing aphids (Martinez-Torres et al. 2001; Jousselin et al. 2009; Nováková et al. 2013; Meseguer et al. 2015, 2017) and psyllids (Hall et al. 2016) phylogenetic relationships, we used up to five *Portiera* genes to reconstruct the phylogeny and divergence of 42 whitefly species belonging to 25 different genera. Using this approach, we found that *Portiera* of *Aleurolobus* and *Singhiella* whitefly species form a monophyletic clade together with *Portiera* of *Bemisia*, the Aleurolobini tribe. Next, we conducted a polymerase chain reaction (PCR) screening to identify two alternative genome rearrangements and the presence/absence of a functional *dnaQ* gene along the obtained *Portiera* phylogeny. Although most screened *Portiera* presented the ancestral gene order and a functional *dnaQ*, all *Portiera* of the Aleurolobini tribe did not seem to encode a copy of *dnaQ* and presented different rearrangements compared with the ancestral order. At the final stage, we sequenced the genome of *Portiera* from *Singhiella simplex*, which is the most basal Aleurolobini species, to corroborate our screening. We found that the genome of *Portiera* from *S. simplex* contains a pseudogenized *dnaQ* and presents a new genome architecture. Also, it presents large intergenic regions and high number of repeat sequences. Finally, we discuss the possible link between the bacteriocyte transmission mode and the appearance of genome instability in *Portiera*.

## Materials and Methods

### Whitefly Collection and Genomic DNA Extraction

A total of 29 samples, accounting for 25 different whitefly species, were obtained from different sources: freshly collected adults (stored in ethanol until use), Prof. Dan Gerling’s ethanol-preserved collection, and *exsiccate* collection samples from the Natural History Museum (NHM) in London (nymphs were removed from dry leaves and sent in ethanol) (supplementary table 1, Supplementary Material online).

Before genomic DNA (gDNA) extractions were performed, five adult insects (or nymphs from the NHM collection) were rehydrated by consecutive passes in 70%, 50%, 30%, and 0% v/v ethanol solutions in sterile water. Whiteflies were transferred to a new 1.5-ml tube containing 80 μl lysis buffer T1 and were homogenized with 1.4-mm zirconia beads (CK14, Bertin Instruments) using a bead-beater (Minilys, Bertin Instruments). gDNA was extracted with NucleoSpin Tissue XS (Macherey-Nagel) following the manufacturer instructions. For the NHM samples, a nondestructive method was used whenever possible. Nymphs were incubated overnight (56 °C) in 80 μl lysis buffer T1 and 8 μl Proteinase K (20 μg/μl). gDNA was extracted from the lysis buffer using the NucleoSpin Tissue XS standard protocol. The nymphs were recovered, cleaned with sterile water, and stored in fresh ethanol. gDNAs from seven samples that had less than five individuals were subjected to whole-genome amplification (GenomiPhi V2, GE Healthcare), following manufacturer instructions, to ensure sufficient material.

For Illumina sequencing, *S. simplex* adults were accidentally collected together with *Pealius mori* adult whiteflies in July 2018 from *Ficus benjamina* (GPS coordinates 31.904511; 34.804562) and stored in ethanol. Later, whiteflies were rehydrated and sexed. Bacteriocytes in adult insects are located in the abdomen, close to the gonads (Buchner 1965). Therefore, female abdomens (50) were dissected under a stereomicroscope using autoclaved 1× phosphate-buffered saline. Abdomens were homogenized with a bead-beater, and gDNA was extracted with NucleoSpin Tissue XS, as described above. Whole-genome shotgun sequencing was performed by NovSeq 6000 using a TruSeq DNA PCR Free Library (2× 150 bp) at Macrogen Europe.

### PCR Screening and Sequencing

To reconstruct *Portiera* phylogeny, five genes present in all insect endosymbionts showing extremely reduced genomes (Moran and Bennett 2014) were selected. These genes are widely used as bacterial phylogenetic markers and include the *16S* and *23S* ribosomal RNAs (rRNAs), the chaperonins *groEL* and *dnaK*, and the RNA polymerase sigma factor *rpoD*. We manually designed *Portiera*-specific universal primers using available *Portiera* genomes from both the Aleyrodinae (*B. tabaci* and *T. vaporariorum*) and Aleurodicinae (*A. dispersus* and *A. floccissimus*) subfamilies in UGENE v1.28.1 (Okonechnikov et al. 2012) (supplementary table 2, Supplementary Material online). Primers melting temperature (*T*_m_), off-targets, and possible primer-dimer interactions were computed with Primer3 software implemented on https://eu.idtdna.com/calc/analyzer (last accessed October 24, 2020).

Primers (0.5 mM each) were mixed with the KAPA2G Robust HotStart ReadyMix (Kapa Biosystems) inside a DNA/RNA UV-Cleaner cabinet (UVC/T-AR). PCR was performed using the following general profile: 95 °C for 5 min, (95 °C for 30 s, *T*_m_ °C for 15 s, 72 °C for 1 min) × 35, 72 °C for 5 min. Annealing temperature (*T*_m_) was set up for each primer set according to Primer3 predictions (supplementary table 2, Supplementary Material online). When required, the temperature was adjusted trying 5 °C above or below of the predicted *T*_m_. PCR product size was confirmed by electrophoresis using 1% agarose gel, purified with DNA Clean & Concentrator 5 (Zymo Research), and sequenced by Sanger technology in both directions at Macrogen Europe. For each amplicon, sequences quality screening/clipping and consensus alignment was performed using the Staden Package (Bonfield and Whitwham 2010).

In parallel, we designed primers that target the DNA polymerase III subunit epsilon *dnaQ*. Also, we targeted two regions with different gene order in *Portiera* of *B. tabaci*, *lepA*-*groEL* (A_*Bt*_) and *secA*-*leuC* (B_*Bt*_), compared with the ancestral gene order found in other sequenced *Portiera*, *groEL*-*rpsA* (A) and *leuC*-*leuD* (B). Primer design and PCRs were conducted as described above using the predicted *T*_m_ (supplementary table 2, Supplementary Material online). PCR products were visualized by electrophoresis using 1% agarose gels. Some obtained amplicons were Sanger sequenced to validate that the correct region was amplified.

To verify species morphological identification, the 5′ region of the mitochondrial cytochrome oxidase 1 (*mtCOI*) gene was amplified and Sanger sequenced, when possible, for each whitefly species collected (analyses are described in supplementary Material and Methods, Supplementary Material online).

### Phylogenetics, Dating, and Ancestral State Reconstruction of *Portiera* Lineages

To infer the phylogenetic relationship and divergence time of *Portiera* from the studied whitefly species, two data sets were used. The first data set incorporated sequences of *Portiera 16S* and *23S* rRNA genes amplified in this study, *16S* and *23S* rRNA gene sequences generated by Thao and Baumann (2004), as well as *16S* and *23S* rRNA gene sequences extracted from downloaded published transcriptomes/genomes (details in the following sections). The final data set contained 59 sequences from 45 different species (including six belonging to the *B. tabaci* species complex). The second data set integrated the sequences of the *16S* and *23S* rRNA genes with those of the three protein coding genes: *dnaK*, *rpoD*, and *groEL*. It contained 32 sequences from 29 whitefly species, mostly obtained in this study plus few that were acquired from public transcriptomes/genomes. Orthologous genes extracted from *Chromohalobacter salexigens* DSM3043 (NC_007963.1) were used as outgroups in the phylogenetic analysis of both data sets (described below).

The *16S* and *23S* rRNA genes were aligned with R-Coffee v11.00.8cbe486 (-mode = rmcoffee -iterate = 100) (Notredame et al. 2000) and pruned with Gblocks v0.91b allowing half of gap positions (-t = d -b5 = h) (Castresana 2000). The three coding genes (*dnaK*, *rpoD*, and *groEL*) were codon aligned with MACSE v2.03 (-prog alignSequences -gc_def 11) (Ranwez et al. 2018) and pruned with Gblocks v0.91b (-t = c -b5 = h). The 19 obtained *mtCOI* gene sequences (5′ region) were aligned in the same way but using the invertebrate mitochondrial code in MACSE v2.03 and no gaps allowed in Gblocks v0.91b. Substitution saturation was assessed using the pruned alignments as an input for Xia’s test implemented in DAMBE v7.2.3 (Xia 2018) (executed under wine v1.6.2-0ubuntu14.2). BEAST v2.5.2 (Bouckaert et al. 2014) was used to infer a Bayesian posterior consensus tree and the divergence time of the different nodes for each of the two data sets outlined above. Detailed procedures of BEAST divergence dating can be found at supplementary Material and Methods, Supplementary Material online.

Results from the *dnaQ* screening were codified as a binary matrix. Then, the binary matrix and the topology of the Bayesian phylogenetic trees were used as input for the Ancestral Character Estimation (ace) function implemented in ape (R package) (R Core Team 2018; Paradis and Schliep 2019). The analyses were conducted twice, using each time the tree that was based on five *Portiera* genes or the three that was based on two *Portiera* genes. The presence of *dnaQ* on the internal nodes of both data sets was estimated using a maximum likelihood approach as a discrete character and a model assuming only gene losses. Phylogenetic trees with *dnaQ* presence probabilities were plotted with ape.

### Whole-Genome Shotgun Sequencing, Genome Assembly, and Annotation of the *S. simplex* and *P. mori* Joint Sample

In order to obtain *Portiera* of *S. simplex* genome, a whole-genome shotgun sequencing strategy was applied. NovaSeq sequencing produced 75,274,888 raw reads that were quality screened with Trimmomatic v0.33 (TruSeq2-PE.fa:2:30:10 LEADING:3 TRAILING:3 SLIDINGWINDOW:4:25 MINLEN:98). Possible polyGs produced by the NovaSeq platform were trimmed with fastp v0.19.7 (-g) (Chen et al. 2018). Cleaned reads were classified with Kraken v2.0.6-beta using a custom database which included several RefSeq genome databases (archaea, bacteria, viral, fungi, and protozoa), all sequenced endosymbionts from whiteflies, the genomes of *B. tabaci* MEAM1 and *Acyrthosiphon pisum*, and all complete mitogenomes of whiteflies. All reads assigned to *Portiera*, *Halomonadaceae*, or *Oceanospirillales* were extracted and assembled with SPAdes v3.13.0 (–sc –careful) (Bankevich et al. 2012). Three contigs larger than 60 kb (385 kb in total) and ∼100× coverage plus several contigs between 80 and 5 kb (420 kb in total) and ∼600× coverage were recovered. Kraken2 classification and coverage suggested two putative *Portiera* populations. To screen for possible *Portiera* other than that of *S. simplex*, all sequences obtained during the PCR screening were used as a query in a BlastN search against the obtained contigs. BlastN results confirmed that two different *Portiera* genomes were present. Large contigs with ∼100× coverage had perfect match to the *Portiera* amplified genes from *P. mori*. Smaller contigs with coverage of ∼600× had perfect match to the amplified *Portiera* genes from *S. simplex*. This confirmed that some *P. mori* individuals were collected together with *S. simplex*, probably due to the ability of both whitefly species to exploit *Ficus benjamina* as a host-tree.

As a result, the Kraken2 database was rebuilt to include the obtained contigs, and cleaned reads were reclassified. *Portiera* reads were reassembled separately according to their whitefly host with SPAdes v3.13.0 (–sc –careful). SSPACE v3 (-k 20 -n 35 -g 3) (Boetzer et al. 2011) and GapFiller v1.10 (-m 50 -o 10 -r 0.6 -n 50 -t 50) (Boetzer and Pirovano 2012) were used for scaffolding and gap-filling the obtained reassembly, respectively. Gap5 from the Staden package was used not only to evaluate the quality of the assemblies but also to detect the presence of chimeras and misassemblies, to join contings manually (when possible), and to check for circular contigs. The first genome to be assembled was that of *Portiera* from *P. mori*. It produced a closed circular contig without requiring any iterative mapping step. In contrast, the *Portiera* genome from *S. simplex* remained as nine contigs after several rounds of iterative mapping, discarding at each round every sequence (if present) with a significant (90% identity threshold) match to the *Portiera* genome of *P. mori*. In brief, iterative mapping was run as follows: Cleaned reads were mapped against the assembled contigs of *S. simplex* with Bowtie v2.3.5.1 (–very-sensitive) (Langmead and Salzberg 2012). Usearch v10 (-usearch_global -query_cov 0.5 -accel 0.5 -strand both -id 0.9) was used do discard reads without a minimum overlap of 50% and 90% identity to the contigs (Edgar 2010). Surviving reads were added to the pool of putative *Portiera* reads from *S. simplex*. The reads were mapped to the contigs with MIRA v4.9.6 (Chevreux et al. 1999), and then imported to Gap5 for manual joining/gap closure. Both final assemblies were corrected with Pilon v1.23 (–fix all, amb) (Walker et al. 2014) and the clean classified reads. Finally, the annotation of the genomes was performed with prokka v1.14.5 (Seemann 2014), using all available *Portiera* genomes for building the protein database of primary annotation (–proteins). Obtained annotations were manually inspected and curated in Artemis v1.5 (Rutherford et al. 2000).

*Singhiella simplex* and *P. mori* mitogenomes assembly and annotation procedures can be found at supplementary Material and Methods, Supplementary Material online.

### *Portiera* Lineages Comparative Genomics

Proteomes of *Portiera* from *S. simplex* (ERZ1272841), *P. mori* (ERZ1272840), *B. tabaci* species—MEAM1 (NC_018677.1), MED (NC_018676.1), and Asia II 3 (NZ_CP016327.1), *T. vaporariorum* (LN649236.1), *A. dispersus* (LN649255.1), and *A. floccissimus* (LN734649.1) were extracted with a custom python script. Orthologous clusters of proteins (OCPs) were calculated with OrthoFinder v2.3.3 (-M msa -S mmseqs -T iqtree) (Emms and Kelly 2019). Obtained OCPs were manually curated based on protein annotations. Shared and specific OCPs were plotted with UpsetR (Conway et al. 2017). Synteny between *Portiera* genomes, based on 230 single-copy core OCPs (from 235), was plotted with genoPlotR (Guy et al. 2010). Finally, metabolic potential comparisons were performed with Pathway Tools v23.5 (Karp et al. 2002).

Curated OCPs were converted into a binary matrix (presence/absence) and species-specific OCPs annotated as hypothetical proteins were discarded (21 OCPs). The binary matrix and the species tree obtained with OrthoFinder v2.3.3 were used as inputs for COUNT v10.04 (Csuos 2010) to reconstruct the gene losses history during *Portiera* evolution. The reconstruction was performed under a posterior algorithm and allowed only gene losses. SEED profiles were computed for each *Portiera* using diamond (BlastP -e 1e-9 -f 100), the nonredundant NCBI database (August 08, 2020 release), and MEGAN6 (Buchfink et al. 2015; Huson et al. 2016).

### Repeats and Intergenic Regions Comparisons, Molecular Evolution Analysis, Transcriptomes Assembly, and Genomic Data Retrieval

Repeats and intergenic regions were compared between *Portiera* lineages and different obligatory endosymbionts to correlate *dnaQ* presence/absence and genome instability. Synonymous (d*S*) and nonsynonymous (d*N*) substitution ratios, nucleotide substitutions per site per year (d*S*/*t* and d*N*/*t*), and omega (*ω*) values were used to compare evolutionary trends in *Portiera* lineages and whitefly mitogenomes. These values were calculated as previously described (Santos-Garcia et al. 2015) using Codeml from PAML v4.7 package (Yang 2007). *Dialeurodes citri* (SRR2856996) and *B. tabaci* SSA1 (SRR5109958) transcriptomes were assembled de novo and several *Portiera* and whiteflies mitochondrial genomes were downloaded to increase our data set. The full procedures of the described analysis can be found in supplementary Material and Methods, Supplementary Material online.

## Results

### Using *Portiera* Gene Sequences to Establish Phylogenetic Relationships and Estimate Divergence Time in Whiteflies

gDNA was extracted from 26 of the 29 collected samples, standing for 22 whitefly species from 17 genera (supplementary table 1, Supplementary Material online). The five sets of primers that target the *Portiera* genes *16S* and *23S* rRNAs, *dnaK*, *rpoD*, and *groEL*, successfully amplified in 25 samples. One sample, *Bemisia reyesi* JHM 7496, was excluded from further analysis because we could not amplify the target regions of the *23S* rRNA and *rpoD* genes. We failed to obtain gDNA from three NHM collection exsiccate samples (supplementary table 1, Supplementary Material online), even when applying a whole-genome amplification approach.

**Fig. 1:**
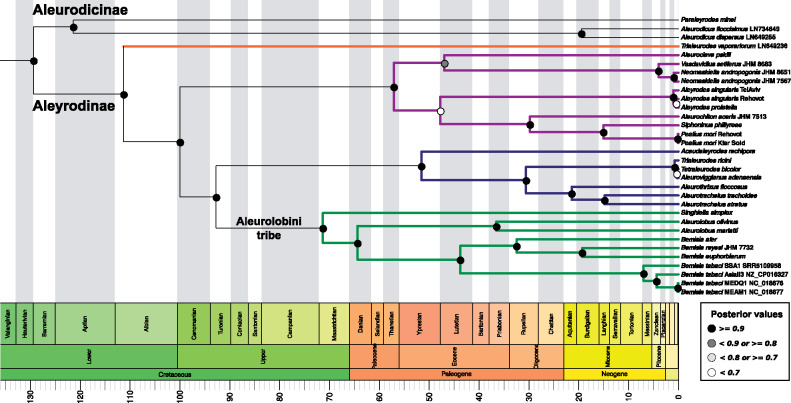
BEAST2-inferred *Portiera* phylogenetic tree (chronogram) based on two rRNA (*16S* and *23S*) and three coding genes (*groEL*, *rpoD*, and *dnaK*) (5G-based tree). Colored branches highlight the four major clades in the Aleyrodinae subfamily. Branch lengths are displayed in million years. Period, Epoch, and Age are according to the geological time scale standards. *Chromohalobacter salexigens* DSM3043 was used as outgroup but is not displayed for plotting reasons.

**Fig. 2:**
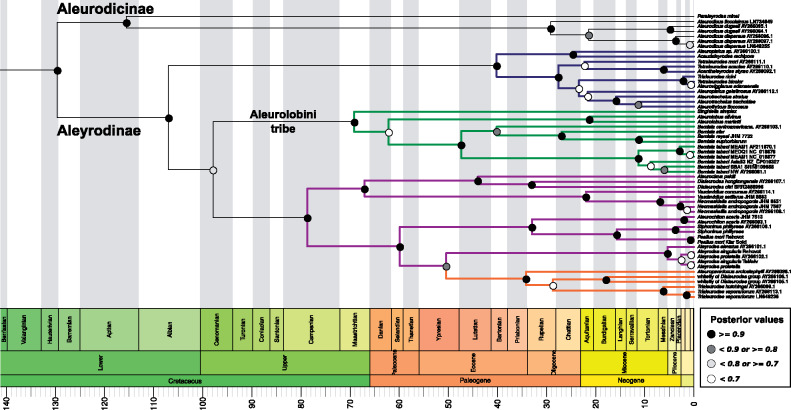
BEAST2-inferred *Portiera* phylogenetic tree (chronogram) based on two rRNA genes (*16S* and *23S*) (2G-based tree). The sequences were generated in this work and in Thao and Baumann (2004). Colored branches highlight the four major clades in the Aleyrodinae subfamily. Branch lengths are displayed in million years. Period, Epoch, and Age are according to the geological time scale standards. *Chromohalobacter salexigens* DSM3043 was used as outgroup but is not displayed for plotting reasons.

Two phylogenetic trees (chronograms) were obtained using two different data sets. One tree was based on the five *Portiera* genes listed above (hereafter 5G-based tree). The second tree was based only on the *16S* and *23S* rRNA genes (hereafter 2G-based tree), which allowed us to include more species in our analyses due to the availability of published sequences (Thao and Baumann 2004). Three main characteristics were common to both trees (figs. 1 and 2): the Aleyrodinae subfamily outcompeted the Aleurodicinae subfamily in the number of analyzed species, the Aleurodicinae was represented by species from the genera *Paraleyrodes* and *Aleurodicus*, and the Aleyrodinae formed four major clusters with similar clustering patterns at the genera level (represented by “green”, “blue”, “purple”, and “orange” colors in figs. 1 and 2).

Some variation between the trees was observed in the “orange” cluster. In the 5G-based tree, the “orange” cluster was found to be the most basal branch and only contained one species, *T. vaporariorum* (fig. 1). In the 2G-based tree, the “orange” cluster (this time containing five species) was integrated within the “purple” cluster and was close to the *Aleyrodes* clade (fig. 2). These topological inconsistencies likely result from the different taxon sampling in the two analyses. The 5G-based tree was well supported and most of the nodes presented posterior values >0.9 (fig. 1). In contrast, the 2G-based tree had a large number of nodes with posterior values below 0.8, especially at some inner branches (fig. 2). Some of the low posterior support values in the 2G-based tree were associated with potential species complexes: *B. tabaci*, *Aleyrodes singularis*/*proletella* and *Neomaskiella andropogonis*. Some inconsistencies in taxonomy were also present in both trees. For example, *Aleuroviggianus adanaensis* was almost identical to *Tetraleurodes bicolor* at the sequence level and some species from the genera *Tetraleurodes*, *Trialeurodes*, and *Dialeurodes* were distributed among different clades.

Among the Aleurodicinae subfamily, *Paraleyrodes minei* was the first species to diverge, around 119.68 Ma (102.74–133.42 95% Highest Posterior Density or HPD) or 112.6 Ma (85.49–133.31 95% HPD) according to the 5G-based or 2G-based trees, respectively (figs. 1 and 2). The divergence of *A. dispersus* from *A. floccissimus* was estimated to be around 20.35 Ma (9.35–33.28 95% HPD) and 30.21 Ma (15.02–47.18 95% HPD) for the 5G-based and 2G-based trees, respectively (figs. 1 and 2). These dates are in agreement with previous estimates Santos-Garcia et al. (2015). In the Aleyrodinae subfamily, despite the topological differences between the two trees, the estimated time of the first cladogenetic event (the first splitting after divergence from the main branch) was similar for the “blue”, “green”, and “purple” clusters. The estimated divergence dates for the “orange” cluster were not comparable between the two data sets. However, if we consider the split between the “green” and “purple”/“orange” clusters in the 2G-based tree as the origin of the lineage leading to *T. vaporariorum*, then, the estimation for *T. vaporariorum* divergence is quite similar: 97.36 Ma (76.14–116.97 95% HPD) in the 2G-based tree and 110.26 Ma (91.43–126.3 95% HPD) in the 5G-based tree. These estimations are in agreement with previous studies (Misof et al. 2014; Santos-Garcia et al. 2015).

The most studied whitefly species, the *B. tabaci* species complex, was part of the “green” cluster in both trees. Our estimations of the emergence time of the *Bemisia* genus and the *B. tabaci* species complex were similar to previous estimations (Santos-Garcia et al. 2015): 44.08 Ma (31.36–57.11 95% HPD) and 7.27 Ma (3.43–11.48 95% HPD) or 47.84 Ma (31.64–64.53 95% HPD) and 11.87 Ma (5.42–19.52 95% HPD), in the 5G-based and 2G-based trees, respectively. Finally, the divergence time between *B. tabaci* species MEAM1 and MED was also in agreement with previous estimates (Santos-Garcia et al. 2015). Taken together, although topological differences existed between the two trees, the convergence of their divergence time estimates supports their robustness.

### Tracking the Origin of Genomic Instability in *Portiera*

**Fig. 3:**
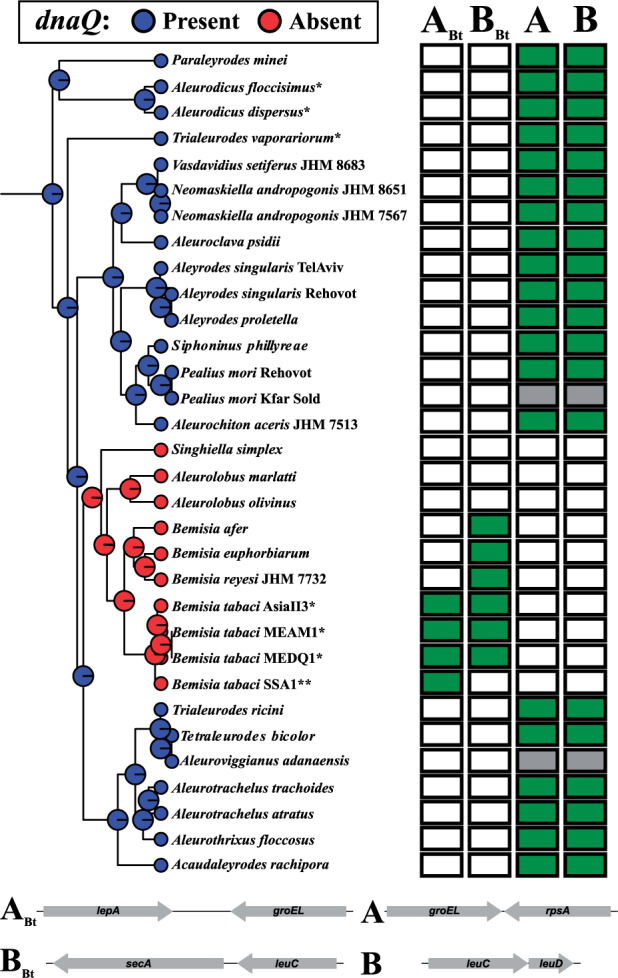
Summary of the screening for the *dnaQ* gene presence or absence and the gene rearrangements. Ancestral state inference was estimated using the *Portiera* 5G-based tree (left). Pie charts at the nodes represent the posterior probability for the presence (blue) or absence (red) of *dnaQ*. Note that all nodes have the probability of 1. The matrix represents the gene rearrangement amplification results (right). The letters above the matrix indicate the four possible rearrangements that were tested. Letters without index refer to the ancestral gene order found in *Portiera* of *Aleurodicus* and *Trialeurodes vaporariorum*. Letters with the subindex *Bt* refer to the gene order found in *Portiera* of *Bemisia tabaci* (bottom). White squares denotes unsuccessful amplifications, green filled squares represent successful amplifications, and gray filled squares indicate that the gene order rearrangements were not tested but the ancestral one (A and B) is assumed. *Full genome available and **no transcripts containing the B_*Bt*_ region were obtained.

*Portiera* of *B. tabaci* lacks the DNA polymerase III proofreading subunit (*dnaQ*). This absence seems to be correlated with the massive rearrangements, large intergenic regions, and repetitive sequences (especially microsatellites) present in the genomes of this *Portiera* lineage (Sloan and Moran 2013; Santos-Garcia et al. 2015). In order to identify the evolutionary time point in which *dnaQ* loss and genome stability appeared, we screened our samples for the presence of *dnaQ* and four possible gene order configurations. We considered the two configurations *groEL*-*rpsA* (A) and *leuC*-*leuD* (B) as ancient because they are shared between *Portiera* from *Aleurodicus* and *T. vaporariorum*. Following this line, we considered the two other configurations, *lepA*-*groEL* (A_*Bt*_) and *secA*-*leuC* (B_*Bt*_), as derived ones because these rearrangements were found so far only in *Portiera* from the *B. tabaci* species complex (fig. 3).

We were able to amplify *dnaQ* of *Portiera* from all species tested except for *Aleurolobus olivinus*, *Ale. marlatti*, *S. simplex*, *B. afer*, *B. euphorbiarum*, and *B. reyesi*. In both the 5G-based and 2G-based trees, these species form a monophyletic clade together with *B. tabaci*, harboring three genera: *Singhiella*, *Aleurolobus*, and *Bemisia*. Based on the ancestral state reconstruction using the 5G-based tree, it is highly likely (posterior probability of 1) that the most recent common ancestor (MRCA) of this clade also lacked a functional *dnaQ* gene (fig. 3). Analysis of the 2G-based tree reached the same prediction with 0.62 posterior probability (supplementary fig. 1, Supplementary Material online). Uncertainty was too large to resolve the presence/absences of *dnaQ* in deeper nodes of the 2G-based tree. Still, following a maximum parsimony scenario, we hypothesize that *dnaQ* is likely to be present in the genome of *Portiera* of all whiteflies, with the exception of the *Singhiella*–*Aleurolobus*–*Bemisia* monophyletic clade.

*Portiera* of all species outside the *Singhiella*–*Aleurolobus*–*Bemisia* clade also presented the ancestral gene order (rearrangements A and B) (fig. 3). *Portiera* of *Bemisia* species outside the *tabaci* species complex only presented the B_*Bt*_ rearrangement, suggesting them to harbor a different rearrangement (than A or ABt) in region A. Although no transcript containing the B_*Bt*_ region was identified in *B. tabaci* SSA1, this species seems to be syntenic to other *B. tabaci* species (supplementary fig. 2, Supplementary Material online). In addition, the fact that the ancestral or modified A and B regions could not be amplified in both of the *Aleurolobus* species and *S. simplex* raises the possibility that several gene rearrangements took place in the A and B regions during the evolution of *Portiera* in the *Singhiella*–*Aleurolobus*–*Bemisia* clade (fig. 3).

### The Genomic and Metabolomic Characterization of *Portiera* from *S. simplex*

To further elucidate the origin of *Portiera* genome instability and its putative effects on functionality, we sequenced the genome of *Portiera* from the most basal species in the *Singhiella*–*Aleurolobus*–*Bemisia* clade, the fig whitefly *S. simplex*. As explained in length in the Materials and Methods section, the sample unintentionally contained individuals of the mulberry whitefly *P. mori*, which shares some host-plants with *S. simplex*. As we were able to classify and recover complete *Portiera* and mitochondrial genomes from both *S. simplex* and *P. mori*, this accidental mixing had not effect on the consequent analyses.

**Fig. 4:**
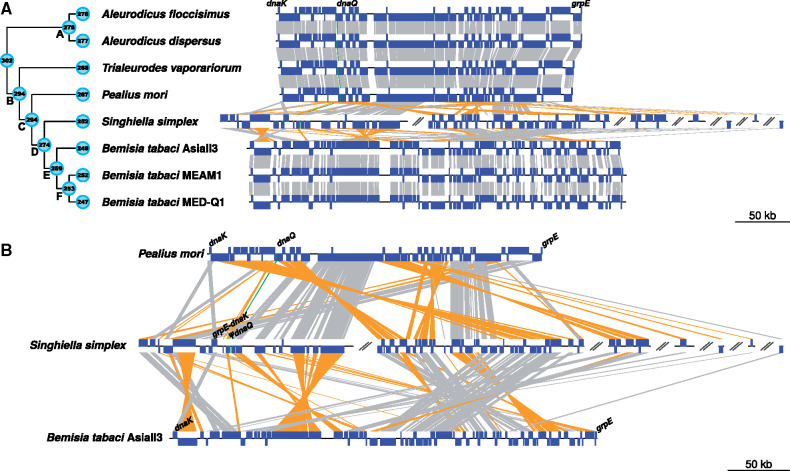
*Portiera* genomes syntenic comparisons based on 230 single-copy core genes. (*A*) Cladogram summarizing *Portiera* phylogenetic relationships based on the species tree obtained as part of the *OrthoFinder* pipeline. Filled circles at the nodes represent the number of coding genes estimated to be present in the MRCA using COUNT. Filled circles at the leaf tips represent the number of coding genes in each *Portiera* genome. Letters at the nodes list the MRCAs to allow comparison with figure 5. *Portiera* genomes are represented linearly. Blue boxes representing syntenic genes in the direct strand (upwards) or in the complementary strand (downwards), gray lines connect genes in the same strand, yellow lines connect genes in different strands, and twisted lines indicate inversions. The green line highlights the position of functional and nonfunctional (*ψ*) *dnaQ* genes. For *Portiera* of *Singhiella simplex*, only contigs containing core genes are represented (seven from nine contigs). (*B*) Magnification of synteny comparisons between *Portiera* of *Pealius mori*, *S. simplex*, and *Bemisia tabaci* AsiaII3.

The genome of *Portiera* from *S. simplex* was recovered as nine contigs (table 1), all ending in repetitive sequences. It is the largest *Portiera* genome described so far, being 134 kb larger than that of *Portiera* from *B. tabaci* (table 1). The number of coding genes was similar in *Portiera* of *S. simplex* and *B. tabaci*, indicating that genome expansion in *Portiera* of *S. simplex* is due to an increase in the size of the intergenic regions, which account for 40% of the genome. The genome of *Portiera* from *S. simplex* presents the lowest coding density (59.6%) and the highest number of direct (23) and inverted (17) repeats among all currently analyzed endosymbionts genomes (table 1). As was already predicted from the PCR amplification and ancestral state reconstruction results, the *dnaQ* gene was found to be nonfunctional (pseudogenized) in *Portiera* of *S. simplex*. The *dnaQ* pseudogene is located in a region that has suffered different rearrangements and an expansion of the intergenic regions (fig. 4*B*). Comparisons to other *Portiera* genomes and different obligatory endosymbionts present in mealybugs, scale insects, and cicadas indicated a clear association between the absence of a functional *dnaQ* and the presence of extended intergenic regions in the endosymbionts’ genomes (supplementary fig. 3, Supplementary Material online; Kruskal–Wallis test, df = 8, *P* value <2.2^e-16^ and pairwise Wilcoxon test with Benjamini–Hochberg FDR).

**Table 1:** General Genomic Features of *Portiera* and Other Endosymbionts Lacking *dnaQ*, Presenting Large Intergenic Regions or Genome Instability

	*Portiera* TeVa	*Portiera* PeMo	*Portiera* SiSi	*Portiera* BeTa	*Uzinura* ASNER	*Tremblaya* PCVAL	*Hodgkinia* TETUND1 and TETUND2	
Accession number	LN649236	LR744089	CACTJB010000000	CP003835	NC_020135	NC_017293	CP007232	CP007233
Host	*T. vaporariorum*	*P. mori*	*S. simplex*	*B. tabaci* MED-Q1	*Aspidiotus nerii*	*Planococcus citri*	*Tettigades undata*	
Genome size (bp)	280,822	277,700	411,975	357,472	263,431	138,931	133,698	140,570
Contigs	1	1	9	1	1	1	1	1
N50 (bp)	NA	NA	17,098	NA	NA	NA	NA	NA
L50	NA	NA	2	NA	NA	NA	NA	NA
GC%	24.69	24.1	26.18	26.12	30.2	58.83	46.77	46.16
Genes^a^	307	308	300	284	275	130	177	184
CDS	268	266	252	247	226	116	121	140
Pseudogenes (CDS)	1	3	11	7	13	19	39	19
CDS avg. length	989.21 ± 675.33	979.95 ± 672.88	977.88 ± 697.31	980.65 ± 697.56	965.30 ± 715.67	622.07 ± 591.77	789.69 ± 700.90	792.58 ± 670.70
CDS avg. GC%	23.88 ± 4.65	23.24 ± 5.01	25.33 ± 4.43	26.35 ± 4.01	29.59 ± 3.34	59.30 ± 3.09	46.15 ± 3.77	45.50 ± 3.81
Intergenic avg. length	62.79 ± 81.43	51.81 ± 63.09	715.44 ± 1,432.48	524.99 ± 871.23	141.63 ± 235.63	173.55 ± 189.19	105.08 ± 140	118.36 ± 164.76
Intergenic avg. GC%	19.12 ± 9.21	17.82 ± 9.37	22.07 ± 8.23	22.40 ± 7.15	23.60 ± 8.41	59.89 ± 6.15	47.53 ± 9.8	46.62 ± 7.46
Coding density (%)	91.46	96.60	59.56	69.60	89.73	81.26	90.49	90.06
Intergenic regions (%)	8.54	3.40	40.44	30.40	10.27	18.74	9.51	9.94
rRNA	3	3	3	3	3	6	3	3
tRNA	34	34	34	33	31	7	13	18
tmRNA	1	1	1	1	1	1	0	0
RnaseP RNA	1	1	1	1	1	0	1	1
*dnaQ*	Yes	Yes	Pseudo	No	No	Yes	Pseudo	Yes
Direct repeats	1	2	23	4	1	3	2	0
Inverted repeats	0	1	17	2	1	4^b^	2	1
Tandem repeats	10	31	3	111	11	0	0	0

a Gene features including pseudogenes.

b Three of them correspond to the duplicated rRNA operons.

Synteny evaluation analysis, based on 230 OCPs (supplementary fig. 4*A*, Supplementary Material online), indicated that *Portiera* of *S. simplex* presents a different genomic architecture when compared both with the ancestral *Portiera* and with the *Portiera* of *B. tabaci* gene order (fig. 4*A*). Nevertheless, a high degree of microsynteny was also observed in some genomic regions.

**Fig. 5:**
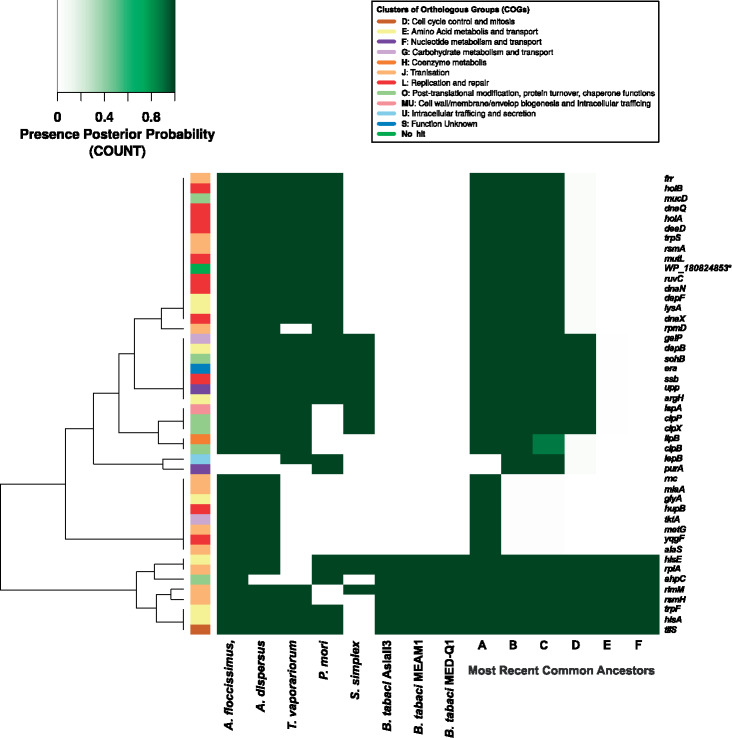
Gene losses in *Portiera* lineages and their MRCAs. Posterior probabilities for the presence of the different genes in the MRCAs were obtained with COUNT. The colored sidebar in the left represents the Clusters of Orthologous Groups (COG) category assigned to each protein encoded by a lost gene. Replication and repair (L category) gene losses are accumulated in the lineage leading to *Singhiella simplex* and *Bemisia tabaci* (MRCA D). MRCAs nodes are the same as in figure 4. The genes (rows) dendrogram was computed using a binary distance and the ward. D2 clustering method. *WP_180824853: NCBI accession number of a hypothetical protein shared between all *Portiera* with the exception of *S. simplex* and *B. tabaci*.

*Portiera* from *S. simplex*, as other sequenced *Portiera*, can synthesize by itself the essential amino acid threonine and the nonessential homoserine. Also, it is able to produce carotenoids, several pyruvate and folate interconversions, and proteins with Fe-S clusters. It requires the aid of the hosting cell to synthesize valine, leucine, isoleucine, phenylalanine, and tyrosine (the enzymes performing the last step of those pathways are encoded by the host), methionine (the precursor homocysteine is provided by the hosting cell), and probably histidine, as previously reported for other *Portiera* lineages (Luan et al. 2015; Santos-Garcia et al. 2015). We found the genome of *Portiera* from *S. simplex* to be metabolically close to that of *B. tabaci* (supplementary fig. 4*B*, Supplementary Material online). Both *Portiera* have lost part of the lysine biosynthetic pathway (fig. 5), which is probably complemented by the hosting cell (Luan et al. 2015). Besides, *Portiera* of *S. simplex* has lost the ability to produce tryptophan (the *trpF* gene is absent), but, in contrast to *Portiera* of *B. tabaci*, can still produce arginine. *Portiera* of *S. simplex* also lacks the aminoacyl-tRNA synthetases *argS*, *asnS*, and *thrS* (lost in all available *Portiera* genomes), *metG* and *alaS* (also lost in *Portiera* of *T. vaporariorum*, *P. mori*, and *B. tabaci*), and *trpS* (lost in *Portiera* of *B. tabaci*) (fig. 5). The tRNA^*Ile*^-lysidine synthetase *tilS*, responsible for avoiding mischarging of methionine instead of isoleucine, was found to be uniquely pseudogenized in *Portiera* of *S. simplex*. In addition, the genome of *Portiera* from *S. simplex* has lost six genes related to the DNA replication and repair machinery (fig. 5). These genes were likely lost, together with other 12 genes, in the MRCA of the *Singhiella*–*Aleurolobus*–*Bemisia* clade (figs. 4*A* and 5).

### Comparative Molecular Evolution among *Portiera* Lineages

We estimated the ratio of synonymous (*S*) and nonsynonymous (*N*) substitutions per site (d*S* and d*N*) and their omega ratio (ω = d*N*/d*S*) in 232 single-copy genes shared among *Portiera* lineages of six whitefly species: *A. dispersus*, *A. floccissimus*, *T. vaporariorum*, *P. mori*, *S. simplex*, and *B. tabaci* (MEAM1). After filtering, 158 orthologous shared genes were kept. To obtain the *S* and *N* per site per year (d*S*/*t* and d*N*/*t*), the values were divided by each lineage divergence time (according to the 5G-based chronogram predictions that presented high internal nodes support): 19.64 Myr for *Aleurodicus*, 111.29 Myr for *Trialeurodes*, 99.98 Myr for *Pealius*, and 71.34 for *Singhiella*–*Bemisia* (fig. 1). d*S*/*t* and d*N*/*t* are normalized values and allow comparisons between lineages (the branch leading to a specific *Portiera* genome).

**Fig. 6:**
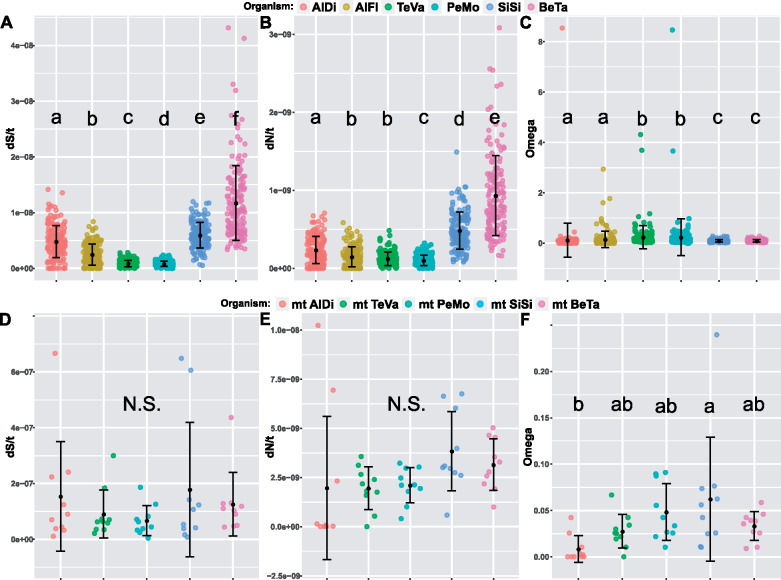
Synonymous (*A*) and nonsynonymous (*B*) substitutions per site per year and their *ω* ratios (*C*) estimated for 158 core shared genes between *Portiera* lineages of six whitefly species. Different letters indicate significant statistical differences between lineages (nonparametric Kruskal–Wallis and Wilcoxon post hoc pairwise tests). Synonymous (*D*) and nonsynonymous (*E*) substitutions per site per year and *ω* ratios (*F*) estimated for ten full mitochondrial genes from six whiteflies species. N.S., no significant difference. Different letters indicate significant statistical differences between lineages (one-way ANOVA and Tukey’s post hoc test). Organism abbreviations are as table 2.

Our analyses indicated that the *Portiera* lineages evolve at different d*S*/*t* (Kruskal–Wallis test, *P* value < 2.2e-16) (fig. 6*A*). *Portiera* of *B. tabaci* was the fastest-evolving lineage, followed by *Portiera* of *S. simplex*, whereas the slowest-evolving lineage was *Portiera* of *P. mori* (table 2). Also, d*N*/*t* values showed statistical differences among *Portiera* lineages (Kruskal–Wallis test, *P* value < 2.2e-16) (fig. 6*B*). Again, *Portiera* of *B. tabaci* and *S. simplex* were the fastest-evolving lineages, whereas *Portiera* of *P. mori* was the slowest-evolving lineage (table 2).

**Table 2:** Average Nucleotide Substitutions per Site per Year and *ω* Ratios for *Portiera* and Mitochondrial Lineages

	Lineage	d*S*/*t*	d*N*/*t*	Omega
*Portiera*	*A. dispersus* (AlDi)	4.79 × 10^−09^	2.35 × 10^−10^	0.1200
	*A. floccissimus* (AlFl)	2.48 × 10^−09^	1.46 × 10^−10^	0.1490
	*T. vaporariorum* (TeVa)	1.02 × 10^−09^	1.43 × 10^−10^	0.2360
	*P. mori* (PeMo)	8.25 × 10^−10^	1.02 × 10^−10^	0.2310
	*S. simplex* (SiSi)	5.94 × 10^−09^	4.85 × 10^−10^	0.0914
	*B. tabaci* (BeTa)	1.17 × 10^−08^	9.32 × 10^−10^	0.0901
Mitochondrion	*Aleurodicus* (mt AlDi)	1.54 × 10^−07^	1.98 × 10^−09^	0.0084
	*T. vaporariorum* (mt TeVa)	1.05 × 10^−07^	2.28 × 10^−09^	0.0276
	*P. mori* (mt PeMo)	6.74 × 10^−08^	2.11 × 10^−09^	0.0484
	*S. simplex* (mt SiSi)	1.79 × 10^−07^	3.84 × 10^−09^	0.0623
	*B. tabaci* (mt BeTa)	1.26 × 10^−07^	3.15 × 10^−09^	0.0333
*Portiera*/mitochondrion	*T. vaporariorum*	103.63	15.97	0.12
	*P. mori*	81.72	20.72	0.21
	*S. simplex*	30.05	7.92	0.68
	*B. tabaci*	10.74	3.38	0.37

The comparison of *ω* ratios, used for testing if the six *Portiera* lineages differ in the selection forces that act on their genomes, resulted in three statistically significant groups (fig. 6*C*, Kruskal–Wallis test, *P* value < 2.2e-16): *Portiera* of *B. tabaci* and *S. simplex* had the lowest *ω* values, *Portiera* of *A. dispersus* and *A. floccissimus* presented intermediate *ω* values, and *Portiera* of *T. vaporariorum* and *P. mori* had the highest *ω* values. Most *ω* values were close to 0 indicating a strong purifying selection force in almost all tested genes. Still, we detected 18 genes presenting signatures of relaxed/adaptive selection (d*S* > 0, ω ≥ 1 and ω ≤ 10) in *Portiera* of *T. vaporariorum* (9 genes), *A. floccissimus* (4), *P. mori* (3), and *A. dispersus* (1) (supplementary table 4, Supplementary Material online). Some of these genes were found to be related to amino acid biosynthesis (*hisH*, *leuC*, *trpC*, and *gatC*), aminoacyl-tRNA synthetases (*sufS* and *cysS*), or to energy metabolism (*atpB* and *cyoD*).

Lastly, we compared between the nucleotide substitution rates in *Portiera* lineages and their insect hosts mitochondria (fig. 6*D*–*F*). Because the mitogenome of *A. floccissimus* is still not available, we calculated the d*S*/*t* and d*N*/*t* values of the *Aleurodicus* lineage using only the mitogenome of *A. dispersus* (dividing the values by 129.35 Ma, the estimated time when the split between the Aleurodicinae and the Aleyrodinae families occurred). Only 12 genes were included in the analysis because mitogenomes annotation was not consistent. The mitochondrial lineages presented nonsignificant d*S*/*t* and d*N*/*t* values and large within lineages variation (one-way analysis of variance [ANOVA], *P* value > 0.2) (fig. 6*D* and *E* and table 2). The *ω* values differed only between *S. simplex* and *A. dispersus* that presented the highest and lowest values, respectively (one-way ANOVA, *P* value < 0.02 and Tukey’s post hoc test) (fig. 6*F*). In all lineages, nearly all *ω* values were below 0.1, indicating a strong effect of purifying selection. Calculation of the d*S*/*t* ratio between the mitochondria and *Portiera* indicated that mitochondrial genomes are evolving prominently faster, with the ratios varying between 10-fold in *B. tabaci* and 100-fold in *T. vaporariorum*. These results agree with previous works showing that mitochondrial genomes from insects present high mutation rates (Song et al. 2012; Allio et al. 2017).

## Discussion

### *Portiera* as a Valuable Resource for Establishing Robust Phylogenetic Relationships in Whiteflies

In contrast to insect groups that rely on adult morphology, the current taxonomy of whiteflies is mostly based on the morphology of one nymphal stage (the puparium). However, this stage presents plasticity in many morphological traits that respond to various abiotic and biotic environmental factors including the identity of the plant host (Manzari and Quicke 2006; Charles 2010), eliminating in many cases the possibility of identifying a definite criterion for classification. This has led to a relatively high number of inaccuracies and misassignments in the group taxonomy (Manzari and Quicke 2006). For example, an extensive cladistic analysis suggested that around half of the 117 Aleyrodinae genera analyzed are not monophyletic (excluding monobasic genera) (Manzari and Quicke 2006). Another study used puparial morphological characters of all 20 Aleurodicinae genera and DNA sequences of nine Aleurodicinae genera, but managed to recover only 60% and 14% of the genera as monophyletic, respectively (Charles 2010). Taking all above in consideration, it is safe to state that whitefly taxonomy can significantly benefit from the development of complementary classification frameworks, especially those using molecular data.

We identified both technical and evolutionary advantages for using *Portiera* gene sequences for inferring the phylogenetic relationships among whiteflies, when compared with other commonly used molecular methods (mainly *mtCOI* gene sequences). First, in contrast to *mtCOI* amplicons, all designed *Portiera* primers had an almost perfect amplification success except for the *rpoD* set that failed to amplify one sample. Second, the specific targeting of *Portiera* genes is by itself a diagnostic tool that allows both differentiating whiteflies from similar insects (e.g., nymphal stages of psyllids) and discriminating between the two main whitefly subfamilies. Discrimination is possible because *Portiera* of the Aleurodicinae subfamily contain two specific insertions in the *23S* rRNA gene (Thao and Baumann 2004). Third, targeting *Portiera* genes is especially useful when studying parasitized samples (supplementary table 1, Supplementary Material online), as the use of universal *mtCOI* primers is, in this case, problematic. Fourth, because *Portiera* is evolving slower than the mitogenome of whiteflies (table 2), its genes usually do not show phylogenetic signal saturation, making them more adequate for solving intergenerative relationships and deeper nodes than the *mtCOI* gene sequences (Coeur d’Acier et al. 2014). On the other hand, it is important to note that *Portiera* gene sequences may be limited in their ability to resolve the relationships in cases of recent speciation events or within species relationships between populations.

### *Portiera* Phylogeny Provides New Insights on the Evolutionary History of Whiteflies

Based on nymphal morphology, the *Singhiella*, *Aleurolobus*, and *Bemisia* genera were reported to be paraphyletic and not closely related (Manzari and Quicke 2006). Moreover, previous studies suggested that the *Singhiella* genus is closer to *Dialeurodes* and unrelated to *Bemisia* (Jensen 2001; Manzari and Quicke 2006). However, the phylogeny of *Portiera* shows that these three genera form a monophyletic clade. Also, *mtCOI* phylogenetic analysis supports the monophyly of this clade (Ovalle et al. 2014; Dickey et al. 2015). We propose that the genera *Singhiella*, *Aleurolobus*, and *Bemisia* belong to the Aleurolobini tribe (see Manzari and Quicke 2006 for a detailed review on whitefly tribes).

An unexpected finding in our analysis was the early origin of the *Paraleyrodes* genus. Originally described as *Aleyrodes*, the nymphal stages of *Paraleyrodes* present typical Aleurodicinae morphological characters, such as subdorsal compound pores or legs with apical claws (Quaintance 1909). However, adults present morphological characters typical of Aleyrodinae, such as small body size and single-vein wings (Quaintance 1909; Martin 1996, 2007). Interestingly, the *Paraleyrodes* genus presents median ocellus, an ancestral character described in Cretaceous taxa (Drohojowska and Szwedo 2015). Our analysis supports the inclusion of the *Paraleyrodes* genus inside the Aleurodicinae subfamily based on its ancient origin and the presence of the *23S* rRNA insertions common to the Aleurodicinae subfamily (Thao and Baumann 2004). Our estimates overlap with the calibration point used, suggesting that the *Paraleyrodes* genus originated in the Lower Cretaceous (100.5–145 Ma). Therefore, *Paraleyrodes* can be considered a long-enduring extant taxon, which may explain the retention of the middle ocellum and the mixture of morphological characteristics of both Aleyrodidae subfamilies. Although speculative, it also could be possible that other hard-to-assign Aleurodicinae genera, such as *Aleuroctarthrus* (presents medium ocellus) and *Palaealeurodicus* (does not present clawed legs), are indeed long-enduring taxa (Martin 2008). These two genera are closely related to *Paraleyrodes* according to cladistic analysis (Charles 2010). Also, *Palaealeurodicus* was placed as basal to all Aleurodicinae based on four mitochondrial genes (Charles 2010). Therefore, *Paraleyrodes* can be considered as sister taxon of *Palaealeurodicus*, which diverged before the radiation of the *Aleurodicus* genus (Charles 2010). Identifying such kind of long-enduring taxa could be an invaluable resource for understanding the evolution of the whitefly superfamily.

### Genome Instability in *Portiera* of the Aleurolobini Tribe

Adaptation to an intracellular lifestyle has a significant impact on bacterial symbionts. Metabolic redundancy between the host and the endosymbiont promotes the dependency of the later on the intracellular environment of the former (Morris et al. 2012). Moreover, vertical transmission drastically reduces the endosymbiont effective population size (*N*_e_) and the chances to acquire new genetic material, eventually leading to the generation of asexual populations. The combined effects of vertical transmission and intracellular lifestyle promote the accumulation of deleterious mutations that are otherwise pruned by selection in larger *N*_e_, which can lead to a massive loss of genes (Moran 1996; Toft and Andersson 2010; Wernegreen 2015). The outcome of the process, known as the Muller’s Ratchet (Moran 1996), is an endosymbiont that harbors a highly reduced genome, with small intergenic regions and very few repetitive sequences (Toft and Andersson 2010; Wernegreen 2015). Common conserved elements include genes that are essential for complementing the host dietary requirements and a minimal machinery for informational flux and translation required for cell maintenance (Moran and Bennett 2014). As a consequence of a reduced or absent replication and recombination machinery, and the minimal presence of repetitive sequences, the genomes of long-standing endosymbionts are almost static (Moran and Bennett 2014). For example, only few inversions were detected in endosymbionts that have been codiverging with their host for more than 100 Myr (Patiño-Navarrete et al. 2013; Chong et al. 2019).

Most *Portiera* lineages do not differ from other long-standing endosymbionts and usually exhibit the “classical” reduced and static genomes (Sloan and Moran 2013; Santos-Garcia et al. 2015). One exception to this “rule” is the genome of *Portiera* from *B. tabaci*, which presents large intergenic regions, extensive rearrangements, and abundance of repetitive sequences (Sloan and Moran 2013; Moran and Bennett 2014; Santos-Garcia et al. 2015). In addition, the genome of *Portiera* from *B. tabaci* presents one of the most reduced sets of DNA replication and repair genes among known long-standing P-endosymbionts, including the loss of the DNA polymerase proofreading subunit (*dnaQ*) (Moran and Bennett 2014). As stated earlier, this loss has been linked to the uncommon extensive genome rearrangements, inversions, abundance of repeated sequences, large intergenic regions, and accelerated evolution found in *Portiera* of *B. tabaci* (Sloan and Moran 2013; Santos-Garcia et al. 2015). Our findings suggest that the massive loss of DNA replication and repair genes is not restricted to *B. tabaci* but is shared by all other members of the *Singhiella*–*Aleurolobus*–*Bemisia* clade (hereafter the Aleurolobini tribe for simplicity). Therefore, it is quite probable that *dnaQ* was already pseudogenized in the last common ancestor of this tribe, more than 70 Ma.

So far, only three genomes displaying long intergenic regions, genome instability, or the lack of functional *dnaQ* have been sequenced from other long-standing endosymbionts: *“Ca.* Uzinura diaspidicola*”*, “*Ca.*Tremblaya princeps”, and *“Ca.* Hodgkinia cicadicola” (Moran and Bennett 2014; Van Leuven et al. 2014; López-Madrigal et al. 2015; Łukasik et al. 2018). Only a single genome of *U. diaspidicola* is currently available, and therefore, it is not clear if the lack of *dnaQ* in this endosymbiont is associated with a significant genome instability. Relative to the genomes of *Portiera* from *B. tabaci* and *S. simplex*, the genome of *U. diaspidicola* presents lower number of repeated sequences and smaller intergenic regions. One explanation to this could be the conservation of the *mutL* gene in *U. diaspidicola* (Moran and Bennett 2014). The enzyme MutL, together with MutS, is part of the mismatch repair system that corrects mismatch events that are produced by base misincorporation and polymerase slippage (Rocha 2003). The genome of *Tre. princeps* presents genome instability signatures such as long intergenic regions, gene conversions, and the presence of several direct/indirect sequence repeats (López-Madrigal et al. 2015). Still, the number of repeats and the length of the intergenic regions are smaller than in *Portiera* of *B. tabaci* or *S. simplex*. The inactivation of the recombination machinery in *Tre. princeps* has been proposed as a strategy to reduce the number of homologous recombination events and their deleterious consequences in highly reduced genomes (López-Madrigal et al. 2015). However, *Tre. princeps* has access to a complementing recombination machinery as it harbors the endosymbiont “Ca. Moranella endobia*”* which has an active recombination machinery (López-Madrigal et al. 2015). For example, *Tre. phenacola* from the mealybug *Phenacoccus peruvianus* presents a chimeric genome that emerged from the fusion with its nested *Sodalis* endosymbiont, a process requiring a recombination machinery (Gil et al. 2018). The presence of a functional *dnaQ* subunit in *Tre. princeps* and the possible access to a complementing recombination machinery suggest that the possible causes of genome instability in *Tre. princeps* are different from those in *Portiera*.

One of the most extreme cases of genome instability was reported in *H. cicadicola*. In some cicada genera, which usually have a long lifespan, *H. cicadicola* has been split into several lineages with different genomic content within the same insect. This enforces functional complementation between the lineages for normal growth (Van Leuven et al. 2014; Łukasik et al. 2018). Although the genomic architecture of *H. cicadicola* seems unstable like that of *Portiera*, there are major differences in the relationship of these two endosymbionts with their hosts. Although *Portiera* is essential for whiteflies, *H. cicadicola* is a coprimary endosymbiont in cicadas and has been replaced several times (Łukasik et al. 2018; Matsuura et al. 2018). Therefore, the selection forces acting on both endosymbionts could be very different: strong purifying selection in the case of *Portiera*, whereas more relaxed, or even nonadaptive selection, in the case of *H. cicadicola* (Łukasik et al. 2018).

Large intergenic regions can allow *Portiera* with unstable genomes to better tolerate rearrangements while the expansion of repeated sequences can increase the chance of deleterious homologous recombination events (Sloan and Moran 2013). Because these *Portiera* show signs of gene conversion and recombination, it can be speculated that long intergenic regions and intergenic repeats are selected in their genomes to increase resilience against deleterious mutations. For example, repeated sequences mostly accumulate at the intergenic regions and pseudogenes of *Portiera* from *B. tabaci* and *S. simplex* suggesting strong purifying selection at the gene level. However, it could be possible that recombination is also counter selected in *Portiera* with unstable genomes. This could explain why *Portiera* lineages within the *B. tabaci* species complex are syntenic after, at least, 7 Myr of divergence and contain a low number of direct/indirect repeats compared with *S. simplex*. In the later, recombination seems still to be active. Therefore, it could be possible that after a period of genome instability and intergenic regions expansion, direct and indirect repeats are counter selected to favor more stable genomes.

In contrast, the location of tandem repeats in *Portiera* of *T. vaporariorum* (8 over 10) and *P. mori* (24 from 31) partially or completely overlap with coding genes. As sequence repeats in coding genes can cause gene inactivation and/or rearrangements, their existence within genes of stable *Portiera* genomes may reflect the presence of a minimal, but functional, DNA repair machinery that allows a more relaxed purifying selection process. In fact, *Portiera* of *B. tabaci* and *S. simplex* showed the lowest *ω* values, indicating stronger purifying selection forces acting on their genomes. Taking together, it is possible that increased resilience combined with a strong purifying selection force at the gene level has helped to maintain *Portiera* in the Aleurolobini tribe (Bennett and Moran 2015).

### The Symbiont or the Egg: Genome Instability and Bacteriocyte Inheritance

Since the beginning of the research on insect symbiosis, it was clear that the whitefly superfamily displays a special mode of transmission of endosymbionts: whole maternal bacteriocytes migrate to the oocyte and enter through the future pedicel (Buchner 1965). In *T. vaporariorum*, *Aleyrodes proletella*, *Aleurodes aceris*, and *Aleurochiton aceris* several bacteriocytes penetrate the oocyte (from five to ten, depending on the species) (Tremblay 1959; Buchner 1965; Szklarzewicz and Moskal 2001). In contrast, in *B. tabaci*, *Bemisia aff. gigantea*, and *Ale. olivinus*, a single bacteriocyte is transmitted (Tremblay 1959; Buchner 1965; Coombs et al. 2007).

Although the phylogenetic relationships of *B. aff. gigantea* are not completely resolved, it is currently considered to be a sister clade of *Aleurolobus* and *B. afer*, and distantly related to *B. tabaci* (Manzari and Quicke 2006). It thus seems that all of the whitefly species with a single-bacteriocyte mode of inheritance belong to one phylogenetic group, the Aleurolobini tribe. A parsimonious explanation might be that the single-bacteriocyte mode of inheritance has evolved in the common ancestor of *Aleurolobus*-*Bemisia*, otherwise we would have to assume that it evolved multiple times in different species: *Ale. olivinus*, *B. aff. gigantea*, and *B. tabaci* (Tremblay 1959; Coombs et al. 2007; Luan et al. 2016, 2018; Xu et al. 2020). It would be interesting to see if the single-bacteriocyte maternal transmission pattern occurs also in *S. simplex*. If it does, it would suggest that the whole Aleurolobini tribe is likely to possess this derived type of bacteriocyte inheritance.

There is an apparent relationship between the emergence of a single-bacteriocyte inheritance mode and the presence of *Portiera* lineages with genomic instability. The single-bacteriocyte inheritance mode could potentially have a considerable impact on *Portiera* evolution because it drastically decreases the effective population size (*N*_e_) compared with the inheritance of multiple bacteriocytes. The extremely low *N*_e_ probably intensified the effect of random genetic drift and accelerated the accumulation of deleterious mutations in *Portiera*. In addition, all the *Portiera* cells that are harbored in the same bacteriocyte are expected to present a homogenized allelic composition because recombination events, if happen, are limited to the bacterial cells inhabiting the same bacteriocyte. This implies a low probability for recovery from a state in which deleterious alleles are formed. At the same time, the single-bacteriocyte inheritance mode also exerts strong purifying selection at both the bacteriocyte and *Portiera* levels each generation as offspring harboring a bacteriocyte or *Portiera* with deleterious mutations will probably suffer from severe fitness costs (Luan et al. 2018). This is somewhat supported by the evidence that extant *Portiera* of the Aleurolobini tribe present moreover a stable gene content, with the massive gene loss events occurring only in their common ancestor. For instance, after ∼70 Myr of divergence, only five and ten genes were lost from the *Portiera* genomes of *S. simplex* and *B. tabaci*, respectively.

Further research on the Aleurolobini tribe is required in order to determine what occurred first: the transition from the multi- to the single-bacteriocyte inheritance mode or the switch from stable to unstable genomic architecture of *Portiera*. In the first case, the evolution of a different mode of transmission could have triggered the DNA replication and repair machinery loss as purifying selection was not able to maintain them under very low *N*_e_ (Lynch 2010). In addition, these losses may have been complemented by an overtake of some of their activities by the genome of the host cell (Santos-Garcia et al. 2014, 2015; Silva and Santos-Garcia 2015; Mao et al. 2018). In the alternative case, we should assume that *Portiera* of the Aleurolobini tribe lost its recombination and repair machinery as a consequence of a continuous genome degradation process (Bennett and Moran 2015). This increased the chances for transmitting *Portiera* with deleterious mutations. A multiple-bacteriocytes inheritance mode results in the transmission of mixtures that can mask the presence of bacteriocytes harboring *Portiera* with deleterious mutations/variations. Instead, if single bacteriocytes are inherited, the *Portiera* presenting deleterious mutations will reduce the fitness of the new-born carrying them and they will be counter selected. Therefore, the evolution of the single-bacteriocyte inheritance mode could have been a compensatory adaptation mechanism of the insect host to exercise an iron grip over *Portiera* transmission for ensuring the viability of its offspring (Campbell et al. 2018).

## Conclusions

Our work brings evidence that gene sequences of the primary endosymbiont “*Candidatus* Portiera aleyrodidarum*”* provide a promising tool for establishing a robust phylogenetic framework of the whitefly superfamily. *Portiera* sequences can be used to establish intergenera relationships, serve as diagnostic tools by themselves, and help in the classification of problematic samples (even parasitized ones). Using the phylogenetic framework, we discovered that whitefly species from the *Singhiella*, *Aleurolobus*, and *Bemisia* genera form a monophyletic tribe, the Aleurolobini. We also found that *Portiera* in all these three genera comprise different genome rearrangements that are uncommon in primary endosymbionts. We suggest that the *Portiera* ancestor of the Aleurolobini tribe suffered a massive DNA replication and repair genes loss, which may have triggered the genomic instability phenomenon. We hypothesize that the appearance of genomic instability is also related to the evolutionary switch made between multi- and single-bacteriocyte mode of inheritance.

## Supplementary Material 

Supplementary data are available at *Genome Biology and Evolution* online.

## Supplementary Material

evaa216_Supplementary_Data

## Notes

Data deposition: Sanger-sequenced *Portiera* and *mtCOI* gene fragments, Novaseq raw reads, and *Portiera* and mitochondrial assembled genomes have been deposited at the European Nucleotide Archive (ENA) under the project number PRJEB31657.
